# A simple powerful bivariate test for two sample location problems in experimental and observational studies

**DOI:** 10.1186/1742-4682-7-13

**Published:** 2010-05-07

**Authors:** Hamed Tabesh, S MT Ayatollahi, Mina Towhidi

**Affiliations:** 1 Department of Biostatistics, Shiraz University of Medical Sciences, Shiraz, Iran; 2 Department of Statistics, Shiraz University, Shiraz, Iran

## Abstract

**Background:**

In many areas of medical research, a bivariate analysis is desirable because it simultaneously tests two response variables that are of equal interest and importance in two populations. Several parametric and nonparametric bivariate procedures are available for the location problem but each of them requires a series of stringent assumptions such as specific distribution, affine-invariance or elliptical symmetry.

The aim of this study is to propose a powerful test statistic that requires none of the aforementioned assumptions. We have reduced the bivariate problem to the univariate problem of sum or subtraction of measurements. A simple bivariate test for the difference in location between two populations is proposed.

**Method:**

In this study the proposed test is compared with Hotelling's *T*^2 ^test, two sample Rank test, Cramer test for multivariate two sample problem and Mathur's test using Monte Carlo simulation techniques. The power study shows that the proposed test performs better than any of its competitors for most of the populations considered and is equivalent to the Rank test in specific distributions.

**Conclusions:**

Using simulation studies, we show that the proposed test will perform much better under different conditions of underlying population distribution such as normality or non-normality, skewed or symmetric, medium tailed or heavy tailed. The test is therefore recommended for practical applications because it is more powerful than any of the alternatives compared in this paper for almost all the shifts in location and in any direction.

## Background

Few medical research studies involve comparing two groups on only a single response variable; comparisons on two or more response variables are usually desired. If a single variable is identified as of major research interest, it will be appropriate to apply a two independent samples t-test or Mann-Whitney test. In some studies, however, two response variables are of equal interest and importance. For example, in studies comparing two different treatments for hypertension, it is equally important to compare their effects on both systolic and diastolic blood pressure. For such studies, a bivariate analysis that compares the treatments on two response variables simultaneously may have advantages over two separate univariate tests, one for each variable. The great advantage of bivariate analysis is the possibility of increased power. If the response variables are not too highly correlated, the bivariate test has a chance of finding significant differences among the treatments even if none of the univariate tests is significant [1].

In most medical research, location analysis may be sufficient and testing the distributions is not necessary. For example, when it is decided to compare two characteristics of a population, such as the weight and height of infants, with those of another population, the researcher tries to compare the bivariate location in two populations. In terms of statistical theory, this problem may be restated as follows.

We consider two independent random bivariate samples

(*x*_1*i*_, *y*_1*i*_), *i *= 1, ..., *m *and (*x*_2*j*_, *y*_2*j*_), *j *= 1, ..., *n *from continuous bivariate populations e.g., weight and height of infants in control and treatment populations. [*X*_1_, *Y*_1_]^' ^and [*X*_2_, *Y*_2_]^' ^denote the joint distributions of (*X*_1_, *Y*_1_*) *and (*X*_2_, *Y*_2_) respectively. We intend to test(1)

against(2)

where ***δ ***= *(δ*_1_, *δ*_2_*) *≠ (0, 0) and *H*_0 _means that the joint distribution of (*X*_1_, *Y*_1_*) *and (*X*_2_, *Y*_2_) are the same.

For the above-mentioned problem, we know that for a bivariate normal population, Hotelling's *T*^2 ^is the best. In addition, under nonsingular linear transformations, *T*^2 ^is invariant.

When the underlying population is unknown, many nonparametric tests have been proposed. In 1958, Blumen [2] described a sign test for the hypothesis that the medians of two or more variables had a particular value for the bivariate case. The slopes of the vector from the bivariate median to the *n *sample points were arranged in ascending order according to the respective angles made with the positive horizontal axis. Blumen's proposed statistic is proportional to the squared distance from the centre of gravity of the hypothesized centre. In 1962, Bennett [3] used certain properties of the multivariate normal integral to develop sign tests for the equality of means in two correlated multivariate populations. Chatterjee and Sen [4] extended the Wilcoxon-Mann-Whitney rank sum test to the case of two variables following a conditional approach. Mardia [5] proposed an unconditional non-parametric statistic using the median vector of the combined sample. Peters and Randles [6] introduced a sign rank affine invariant test for the difference in location between two elliptically symmetric populations. Hettmansperger and Oja [7] developed a multivariate invariant sign-test for the multi-sample location problem. Sen and Mathur [8] used the angles made by centerized data for two samples with the positive direction of the *x*-axis to construct a test statistic suggested as an affine-invariant test statistic for the bivariate two sample location problem. Sen and Mathur [9] proposed a consistent test similar to the Mann-Whitney test for difference in locations between two bivariate populations. LaRocque et al. [10] extended the univariate Wilcoxon sign rank test to the bivariate location problem. Baringhaus and Franz [11] proposed a test statistic using the difference between the sums of all the Euclidean interpoint distances. Mathur [12] suggested a nonparametric bivariate test for two sample location problem that did not require affine-invariance or elliptic-symmetry to be assumed.

The findings of most of these tests are not easy to apply and their powers depend on the direction of shifts and the covariance matrix of the alternative distribution. Some of the proposed tests are powerful only for particular forms of distributions and some of them require specific assumptions to verify the test statistics. Thus, it seems that the tests available in the literature are not wholly adequate and hence it is necessary to introduce a test statistic more powerful than the existing ones, which does not depend on the covariance structure of the underlying population and is also easy to apply with readily available software for those who are not experts in statistics.

In the following section, we present a simple bivariate test statistic for the two sample location problem. To investigate the power of the proposed test and to compare it with the alternatives in the literature, a simulation study was carried out. A summary of the power study is displayed in the results and discussion sections. In the conclusion section, an application of the proposed test statistic to a real set of data is given.

## Methods

### Test Statistic

Let (*X*_1*i*_, *Y*_1*i*_) *i *= 1, ..., *m *and (*X*_2*j*_, *Y*_2*j*_) *j *= 1, ..., *n *be two independent random samples from bivariate populations. [*X*_1 _*Y*_1_]^' ^and [*X*_2 _*Y*_2_]^' ^denote the joint distributions of *X*_1_, *Y*_1 _and *X*_2_, *Y*_2 _respectively. We intend to test the null hypothesis given in (1) against the alternative (2). According to the structure of this testing problem, it is presumed that the two distributions [*X*_1 _*Y*_1_]^' ^and [*X*_2 _*Y*_2_]^' ^have the same structural form, but there may be a location shift in [*X*_2 _*Y*_2_]^' ^with respect to [*X*_1 _*Y*_1_]^'^. We therefore aim to test the existence of a location shift.

It is obvious that many tests are available to test the difference in locations between two univaraite populations. It is therefore desirable to find a convenient transformation for changing the bivariate data to the univariate case. We implement our test for three possible combinations of shift direction as follows:

(i) When the shift directions for two variables are the same i.e. δ_1 _δ_2 _> 0, random variables are defined as *S*_1*i *_= *X*_1*i *_+ *Y*_1*i *_for *i *= 1, ..., *m *and *S*_2*j *_= *X*_2*j *_+ *Y*_2*j *_for *j *= 1, ..., *n*. Now to test the null hypothesis *H*_0 _in (1) against *H*_*A *_in (2), it is sufficient to test

against

where δ = δ_1 _+ δ_2 _is a location difference parameter. In fact, this is a location problem in the univariate case and an available test such as the Mann-Whitney test can be used to solve it.

(ii) When the shift directions for two variables are not the same i.e., δ_1 _δ_2 _< 0, the random variables are defined as  for *i *= 1, ..., *m *and  for *j *= 1, ..., *n*. Therefore, it is sufficient to test

against

Where δ = δ_1 _- δ_2 _is a location parameter. For this location problem in the univariate case, the Mann-Whitney test is again used.

(ii) When (δ_1 _= 0, δ_2 _≠ 0) or (δ_1 _≠ 0, δ_2 _= 0), it is enough to apply a rank test to the second variable or the first variable, respectively.

**Remark 1: **To decide which of the above three methods must be used in practice, first the values  and  are computed, where ,  and , . Then if  > 0, method (i) is used and if  < 0, it is appropriate to use method (ii); and method (iii) is used for the testing problem when () or ().

**Remark 2**: (a) Note that, when the two variables are on significantly different scales, the data have to be transferred by the following relations before solving the testing problem:

and

where ,  and  and  are the standard deviations of the random variables *X*_1 _(or *X*_2_) and *Y*_1 _(or *Y*_2_), respectively.

(b) In application, when the two variables are on significantly different scales, the data are transformed by the following relations before using a test statistic and testing hypotheses:

and

where ,  and , are the samples pooled variances of the first variables and the second variables in (*X*_1_, *Y*_1_) and (*X*_2_, *Y*_2_), respectively.

### Power

This section indicates the results of a Monte Carlo study to assess the power of the new test. For comparison purposes, the performances of the following tests were simulated:

(1) Hotelling's *T*^2 ^test with test statistic:

where *S*^-1 ^is the inverse of the sample variance-covariance matrix S [13].

(2) The Rank test, which is based on marginal ranks, is given by

where *N *= *m *+ *n*, *i *= 1, ..., *N*, *j *= 1,2,  are the set of scores for each *j *= 1,2 and *X*_*ij *_are independent identically-distributed random variables with a continuous bivariate distribution [14].

(3) The Cramer test with test statistic:

where the function ϕ is the kernel function.  is recommended for location alternatives [11].

(4) The Mathur's test based on the test statistic:

where ,  and  for *i *= 1, ..., *m j *= 1, ..., *n *[12].

The new proposed test (P) was compared with the above four tests using samples from bivariate normal and non-normal distributions. Simulations were run for bivariate normal with ρ = -0.5,0,0.5. Also, simulations were run for some non-normal distributions generated using the *g*-and-*h *distribution [15], i.e. generating *Z*_*ij *_from a bivariate normal distribution and setting .

For *g *= 0 this expression is taken to be .

As the *g*-and-*h *distribution provides a convenient method for considering a very wide range of situations corresponding to both symmetric and highly asymmetric distributions, its use is highly recommended. The case *g *= *h *= 0 corresponds to a normal distribution, the case *g *= 0 corresponds to a symmetric distribution, and, as g increases, the skewness increases as well. For example, with *g *= 0.5 and *h *= 0, the skewness is 1.75, which is great [16].

In this study, simulations were run with *g *= 0.25 and *g *= 0.5 to span the range of skewness values that seems to occur in practice.

The parameter h determines the heaviness of the tail. As h increase, the heaviness increases as well. With *h *= 0.2 and *g *= 0, the kurtosis equals 36. This might seem extreme, but even higher values were found by Wilcox, so our simulations were run for *h *= 0.2 [16].

## Results and discussion

The results in Table 1, Table 2, Table 3, Table 4, Table 5 and Table 6 were based on 10,000 samples of sizes 15, 18 from a bivariate population with location parameters (δ_1_, δ_2_). A nominal significance level of 0.05 was used. STAT, MASS, CRAMER and ICSNP libraries in R program version 2.10.0 were used.

**Table 1:** Monte Carlo rejection proportion for the bivariate normal population (ρ = 0), m = 15, n = 18

δ_1_, δ_2_	P	*T*^2^	R	C	M
0.00,0.00	0.0500	0.0511	0.0539	0.0538	0.0485
0.15,0.15	0.0878	0.0757	0.0782	0.0791	0.0529
0.15,0.25	0.1197	0.1001	0.1021	0.1034	0.0581
0.25,0.25	0.1589	0.1271	0.1289	0.1320	0.0636
0.30,0.40	0.2653	0.2160	0.2095	0.2202	0.0840
0.40,050	0.3950	0.3275	0.3189	0.3333	0.1143
0.60,0.70	0.6915	0.6069	0.5911	0.6072	0.2293
0.80,0.90	0.8951	0.8414	0.8216	0.8443	0.4327
1.10,1.30	0.9958	0.9913	0.9874	0.9912	0.8408
1.60,1.80	1.0000	0.9999	0.9999	0.9999	0.9982
					
					

0.00,0.00	0.0477	0.0511	0.0539	0.0538	0.0485
-0.15,0.15	0.0831	0.0748	0.0782	0.0756	0.0521
0.15,-0.25	0.1131	0.1060	0.1025	0.1062	0.0594
-0.25,0.25	0.1506	0.1240	0.1214	0.1235	0.0646
0.30,-0.40	0.2581	0.2118	0.2071	0.2159	0.0835
-0.40,050	0.4028	0.3254	0.3166	0.3259	0.1150
0.60,-0.70	0.6959	0.6105	0.5873	0.6106	0.2322
-0.80,0.90	0.8995	0.8477	0.8271	0.8507	0.4331
1.10,-1.30	0.9954	0.9905	0.9880	0.9907	0.8446
-1.60,1.80	1.0000	1.0000	1.0000	1.0000	0.9980

**Table 2:** Monte Carlo rejection proportion for the bivariate normal population (ρ = 0.5), m = 15, n = 18

δ_1_, δ_2_	P	*T*^2^	R	C	M
0.00,0.00	0.0477	0.0511	0.0518	0.0532	0.0474
0.15,0.15	0.0768	0.0676	0.0692	0.0755	0.0516
0.15,0.25	0.0995	0.0861	0.0891	0.0984	0.0535
0.25,0.25	0.1245	0.0997	0.1021	0.1220	0.0585
0.30,0.40	0.1927	0.1608	0.1605	0.1956	0.0726
0.40,050	0.2912	0.2355	0.2345	0.2841	0.0973
0.60,0.70	0.5170	0.4431	0.4376	0.5176	0.1764
0.80,0.90	0.7468	0.6677	0.6632	0.7440	0.3320
1.10,1.30	0.9606	0.9357	0.9280	0.9608	0.7130
1.60,1.80	0.9996	0.9988	0.9985	0.9993	0.9848
1.90,2.20	1.0000	0.9998	0.9999	0.9999	0.9998
					
					

0.00,0.00	0.0481	0.0511	0.0518	0.0532	0.0474
-0.15,0.15	0.1221	0.1016	0.0958	0.0750	0.0545
0.15,-0.25	0.1874	0.1546	0.1453	0.1034	0.0596
-0.25,0.25	0.2608	0.2082	0.1930	0.1265	0.0717
0.30,-0.40	0.4688	0.3829	0.3503	0.2289	0.0996
-0.40,050	0.6704	0.5954	0.5446	0.3785	0.1464
0.60,-0.70	0.9337	0.8974	0.8564	0.7372	0.3192
-0.80,0.90	0.9968	0.9896	0.9819	0.9504	0.5962
1.10,-1.30	1.0000	1.0000	0.9999	0.9997	0.9459

**Table 3:** Monte Carlo rejection proportion for the bivariate normal population (ρ = -0.5), m = 15, n = 18

δ_1_, δ_2_	P	*T*^2^	R	C	M
0.00,0.00	0.0481	0.0511	0.0539	0.0526	0.0474
0.15,0.15	0.1227	0.1033	0.1016	0.0759	0.0542
0.15,0.25	0.1874	0.1528	0.1442	0.0988	0.0602
0.25,0.25	0.2671	0.2145	0.1945	0.1257	0.0685
0.30,0.40	0.4688	0.3896	0.3520	0.2284	0.0992
0.40,050	0.6774	0.5876	0.5438	0.3834	0.1448
0.60,0.70	0.9337	0.8952	0.8565	0.7368	0.3176
0.80,0.90	0.9947	0.9916	0.9826	0.9481	0.5977
1.10,1.30	1.0000	0.9999	0.9999	0.9998	0.9449
					
					

0.00,0.00	0.0477	0.0511	0.0539	0.0526	0.0474
-0.15,0.15	0.0768	0.0648	0.0688	0.0727	0.0516
0.15,-0.25	0.0928	0.0911	0.0902	0.0974	0.0553
-0.25,0.25	0.1245	0.0976	0.0990	0.1118	0.0585
0.30,-0.40	0.1902	0.1574	0.1591	0.1901	0.0743
-0.40,050	0.2912	0.2335	0.2300	0.2791	0.0959
0.60,-0.70	0.5281	0.4348	0.4325	0.5178	0.1823
-0.80,0.90	0.7468	0.6747	0.6705	0.7459	0.3300
1.10,-1.30	0.9567	0.9371	0.9295	0.9612	0.7141
-1.60,1.80	0.9996	0.9994	0.9988	0.9999	0.9847
1.90,-2.20	1.0000	1.0000	1.0000	1.0000	0.9998

**Table 4:** Monte Carlo rejection proportion for the bivariate skewed population, m = 15, n = 18

δ_1_, δ_2_	P	*T*^2^	R	C	M
0.00,0.00	0.0492	0.0520	0.0539	0.0534	0.0486
0.15,0.15	0.0863	0.0729	0.0787	0.0771	0.0494
0.15,0.25	0.1181	0.0951	0.1044	0.1010	0.0514
0.25,0.25	0.1533	0.1218	0.1314	0.1276	0.0554
0.30,0.40	0.2591	0.2021	0.2137	0.2096	0.0689
0.40,050	0.3895	0.3162	0.3289	0.3222	0.0943
0.60,0.70	0.6730	0.5727	0.6000	0.5954	0.1922
0.80,0.90	0.8772	0.8095	0.8248	0.8335	0.3858
1.10,1.30	0.9926	0.9832	0.9861	0.9900	0.8220
1.60,1.80	0.9999	0.9997	0.9999	0.9998	0.9975
1.90,2.20	1.0000	1.0000	1.0000	1.0000	0.9998
					
					

0.00,0.00	0.0485	0.0520	0.0539	0.0534	0.0486
-0.15,0.15	0.0818	0.0724	0.0788	0.0740	0.0534
0.15,-0.25	0.1143	0.1012	0.1034	0.1053	0.0597
-0.25,0.25	0.1467	0.1173	0.1240	0.1224	0.0669
0.30,-0.40	0.2514	0.2015	0.2124	0.2091	0.0870
-0.40,050	0.3919	0.3099	0.3274	0.3171	0.1146
0.60,-0.70	0.6773	0.5770	0.5976	0.5988	0.2376
-0.80,0.90	0.8821	0.8147	0.8352	0.8406	0.4301
1.10,-1.30	0.9921	0.9824	0.9876	0.9887	0.8268
-1.60,1.80	1.0000	1.0000	1.0000	1.0000	0.9962

**Table 5:** Monte Carlo rejection proportion for the bivariate highly skewed population, m = 15, n = 18

δ_1_, δ_2_	P	*T*^2^	R	C	M
0.00,0.00	0.0487	0.0470	0.0539	0.0532	0.0500
0.15,0.15	0.0834	0.0656	0.0834	0.0725	0.0455
0.15,0.25	0.1128	0.0836	0.1086	0.0929	0.0472
0.25,0.25	0.1478	0.1058	0.1398	0.1175	0.0501
0.30,0.40	0.2464	0.1734	0.2314	0.1942	0.0589
0.40,050	0.3664	0.2661	0.3520	0.2948	0.0777
0.60,0.70	0.6264	0.4938	0.6243	0.5627	0.1694
0.80,0.90	0.8351	0.7194	0.8386	0.8062	0.3500
1.10,1.30	0.9779	0.9429	0.9834	0.9853	0.7959
1.60,1.80	0.9991	0.9973	0.9998	0.9997	0.9933
1.90,2.20	1.0000	0.9997	1.0000	1.0000	0.9994
					
					

0.00,0.00	0.0480	0.0470	0.0539	0.0532	0.0500
-0.15,0.15	0.0796	0.0647	0.0813	0.0738	0.0531
0.15,-0.25	0.1095	0.0885	0.1088	0.0987	0.0643
-0.25,0.25	0.1412	0.1024	0.1335	0.1119	0.0657
0.30,-0.40	0.2363	0.1738	0.2270	0.1938	0.0945
-0.40,050	0.3671	0.2640	0.3479	0.2924	0.1152
0.60,-0.70	0.6272	0.4906	0.6294	0.5638	0.2400
-0.80,0.90	0.8419	0.7280	0.8437	0.8110	0.4222
1.10,-1.30	0.9812	0.9425	0.9841	0.9830	0.7890
-1.60,1.80	0.9997	0.9974	0.9998	1.0000	0.9882
1.90,-2.20	1.0000	0.9998	1.0000	1.0000	0.9980

**Table 6:** Monte Carlo rejection proportion for the bivariate heavy tailed population, m = 15, n = 18

δ_1_, δ_2_	P	*T*^2^	R	C	M
0.00,0.00	0.0505	0.0470	0.0539	0.0523	0.0476
0.15,0.15	0.0754	0.0598	0.0723	0.0717	0.0535
0.15,0.25	0.0945	0.0720	0.0898	0.0890	0.0563
0.25,0.25	0.1190	0.0854	0.1091	0.1062	0.0607
0.30,0.40	0.1928	0.1379	0.1725	0.1684	0.0742
0.40,050	0.2789	0.1993	0.2570	0.2465	0.0969
0.60,0.70	0.4974	0.3782	0.4731	0.4554	0.1707
0.80,0.90	0.7167	0.5752	0.6953	0.6823	0.3050
1.10,1.30	0.9369	0.8581	0.9366	0.9326	0.6325
1.60,1.80	0.9977	0.9835	0.9985	0.9983	0.9407
1.90,2.20	0.9998	0.9976	0.9999	0.9999	0.9904
					
					

0.00,0.00	0.0480	0.0470	0.0539	0.0523	0.0476
-0.15,0.15	0.0718	0.0579	0.0722	0.0671	0.0500
0.15,-0.25	0.0921	0.0774	0.0905	0.0925	0.0573
-0.25,0.25	0.1154	0.0852	0.1059	0.0999	0.0596
0.30,-0.40	0.1849	0.1359	0.1732	0.1642	0.0750
-0.40,050	0.2743	0.2004	0.2538	0.2373	0.0980
0.60,-0.70	0.4986	0.3694	0.4717	0.4521	0.1720
-0.80,0.90	0.7236	0.5840	0.7053	0.6866	0.3011
1.10,-1.30	0.9394	0.8595	0.9373	0.9346	0.6351
-1.60,1.80	0.9965	0.9847	0.9980	0.9987	0.9436
1.90,-2.20	0.9999	0.9972	0.9999	1.0000	0.9905

Under the bivariate normal distribution with different correlations, the simulation results showed that the proposed test statistic performed better than any of the test statistics compared here for almost all shifts in location.

The findings of this study show that the proposed test had greater power than Hotelling's *T*^2 ^and Mathur's test for skewed populations. Also, had greater power than Cramer's test for a small shift in location but reached a power level equivalent to that of the Rank test for a skewed population.

When the population was highly skewed, the proposed test statistic dominated Hotelling's *T*^2 ^and Mathur's test for almost all shifts in location. It also dominated Cramer's test for small and moderate shifts in location.

The power of the proposed test was greater than any of its competitors for almost all shifts in location except the Rank test for a large shift in location under a heavy tailed bivariate distribution.

The simulation results revealed that the proposed test statistics would perform much better when the underlying population was bivariate normal, skewed, highly skewed or heavy tailed.

The simulations were done for sample sizes m = 10 and n = 10, and the results were closely similar. In general, simulations performed for different sample sizes showed similar power trends.

## Conclusions

In the medical field, where two measurements such as changes in closing volume and white blood cell count [18], cholesterol level and blood pressure, potassium and sodium [19] are considered for important diagnoses, the bivariate values may be related in an unknown way, so bivariate analysis is considered an important problem. The population bivariate distributions may be unknown in many cases so parametric tests cannot be applied. Some nonparametric tests require assumptions that are hard to validate. The proposed test does not require the stringent assumption of affine-invariance or elliptic-symmetry, and it is very easy to understand and apply using only regular statistical programs. In fact, we have solved the bivariate problem by reducing it to the univariate problem of sum or difference of measurements.

The results of the simulation studies showed that the proposed test performed better than most of it competitors for almost all the shifts in location. This very important property of the proposed test statistic established that it would perform much better whether the underlying population was normal or non-normal, skewed or symmetric, medium tailed or heavy tailed. Therefore, its application is recommended, since it is more powerful than any of the alternatives compared here for almost all shifts in location and in any direction.

Most of the test statistics available in the literature were difficult to compute even with the help of the computer. The proposed test statistic could easily be calculated manually for small and moderate sized data sets, which is another important property.

Here for illustration, the application of the proposed test statistic to a real data set is given. Ayatollahi [17] studied growth velocity standards from longitudinally measured infants aged 0-2 years born in Shiraz. A cohort of 317 healthy neonates were selected and followed for two years. They were visited at home at different target ages and several variables were measured. Here the researchers focused on 12 months old children, and we interested in two dependent variables, height and weight, and a grouping variable, mother's education level. Ages were recorded exactly on the basis of the difference between the date of visit and date of birth in days, and then converted to months. The weight velocity over the first year of life was defined as the difference between weight at 12 months old and weight at birth divided by the difference between date of visit at 12 months old and date of birth [17].

Simultaneously, comparison of weight and height velocities between two groups of infants, with primary and secondary educated mothers, was the main interest. The bivariate observations were the 87 measurements on weight velocity (*X*_1_) and height velocity (*Y*_1_) over the first year of life for infants with primary educated mothers and 54 measurements on weight velocity (*X*_2_) and height velocity (*Y*_2_) over the first year of life for infants with secondary educated mothers.

Table 7 shows  kg/mo,  kg/mo,  cm/mo and  cm/mo. Computing  and , we found that  and , hence  > 0. According to the sign of , method (i) should be used. So *S*_*i *_= *X*_*i *_+ *Y*_*i *_were computed for infants with primary and secondary educated mothers (*i *= 1,2). The Mann-Whitney test was used to test *H*_0_: [*X*_1 _+ *Y*_1_] ~ [*X*_2 _+ *Y*_2_] against *H*_*A*_: [*X*_1 _+ *Y*_1_] ~ [*X*_2 _+ *Y*_2 _+ δ]; the p-value = 0.006 led to rejection of the null hypothesis at the 5% level of significance. This was consistent with the conclusion reached using Hotelling's *T*^2 ^test for the same data set with p-value = 0.027.

**Table 7:** Mean and standard deviation of weight and height velocity of infants over the first year of life with primary and secondary educated mothers (m = 87, n = 54)

Mother education		Weight velocity	Height velocity
Primary (m = 87)	Mean	0.483	2.002
	Std. Deviation	0.087	0.209

Secondary (n = 54)	Mean	0.497	2.094
	Std. Deviation	0.088	0.195

In order to illustrate the performance of the proposed test versus Hotelling's *T*^2 ^test especially for small size samples, a random sample of 22 infants was selected. Weight and height velocities for this random sample, a part of data from Ayatollahi (2005) [17], are presented in Table 8. The bivariate observations were the 13 measurements on weight velocity (*X*_1_) and height velocity (*Y*_1_) over the first year of life for infants with primary educated mothers and 9 measurements on *X*_2_, *Y*_2 _for infants with secondary educated mothers. In Table 9, mean and standard deviation of weight and height velocity over the first year of life for infants with primary and secondary educated mothers are presented. Using the proposed test, the p-value was 0.030, which led to rejection of the null hypothesis at the 5% level of significance. However, this was not consistent with the conclusion reached using Hotelling's *T*^2 ^test (p-value = 0.072). In this small data set, Hotelling's *T*^2 ^could not detect the difference, but the proposed test could detect it as well as in the large data set.

**Table 8:** Data from the weight and height velocity of infants over first year of life with primary and secondary educated mothers

Infants with primary educated mothers		Infants with secondary educated mothers	
**Weight velocity**	**Height velocity**	**Weight velocity**	**Height velocity**

0.49	2.00	0.53	2.04
0.30	1.42	0.58	2.25
0.48	1.90	0.45	2.05
0.53	1.92	0.61	1.98
0.51	2.01	0.45	1.87
0.48	1.90	0.41	2.23
0.43	1.95	0.54	2.27
0.40	2.07	0.44	2.23
0.67	2.11	0.44	2.23
0.62	2.28		
0.42	2.03		
0.52	1.89		
0.42	1.86		

**Table 9:** Mean and standard deviation of weight and height velocity of infants over first year of life with primary and secondary educated mothers (m = 13, n = 9)

Mother education		Weight velocity	Height velocity
Primary (m = 13)	Mean	0.483	1.950
	Std. Deviation	0.095	0.196

Secondary (n = 9)	Mean	0.493	2.128
	Std. Deviation	0.072	0.146

## Competing interests

The authors declare that they have no competing interests.

## Authors' contributions

HT proposed the test, most of the redaction, simulation study, application to weight and height velocity. SMTA conceptualized and supervised the study. MT proposed the test and redaction. All authors read and approved the final manuscript.

## Authors' information

Corresponding author: SMT Ayatollahi, Ph.D., FSS, C.Stat. Professor of Biostatistics, The Medical School, Shiraz University of Medical Sciences, Shiraz, Islamic Republic of Iran. P.O.Box 71345-1874
